# Comparative Transcriptomic Analysis Provides Novel Insights into the Blanched Stem of *Oenanthe javanica*

**DOI:** 10.3390/plants10112484

**Published:** 2021-11-17

**Authors:** Sunjeet Kumar, Xinfang Huang, Gaojie Li, Qun Ji, Kai Zhou, Guopeng Zhu, Weidong Ke, Hongwei Hou, Honglian Zhu, Jingjing Yang

**Affiliations:** 1 The State Key Laboratory of Freshwater Ecology and Biotechnology, The Key Laboratory of Aquatic Biodiversity and Conservation of Chinese Academy of Sciences, Institute of Hydrobiology, Chinese Academy of Sciences, Wuhan 430072, China; kumar.sunjeet082@gmail.com (S.K.); ligaojie@ihb.ac.cn (G.L.); houhw@ihb.ac.cn (H.H.); 2 University of Chinese Academy of Sciences, Beijing 100049, China; 3 Key Laboratory for Quality Regulation of Tropical Horticultural Crops of Hainan Province, Engineering Research Center of the Ministry of Education for New Variety Breeding of Tropical Crop, School of Horticulture, Hainan University, Haikou 570228, China; guopengzhu@163.com; 4 Institute of Vegetables, Wuhan Academy of Agricultural Sciences, Wuhan 430207, China; huangxinfang99@163.com (X.H.); jiqun741@sina.com (Q.J.); 13607157304@163.com (K.Z.); wdke63@163.com (W.K.)

**Keywords:** blanching, transcriptome, photosynthesis, plant hormones, transcription factors

## Abstract

In the agricultural field, blanching is a technique used to obtain tender, sweet, and delicious water dropwort stems by blocking sunlight. The physiological and nutritional parameters of blanched water dropwort have been previously investigated. However, the molecular mechanism of blanching remains unclear. In the present study, we investigated transcriptomic variations for different blanching periods in the stem of water dropwort (pre, mid, post-blanching, and control). The results showed that many genes in pathways, such as photosynthesis, carbon fixation, and phytohormone signal transduction as well as transcription factors (TFs) were significantly dysregulated. Blanched stems of water dropwort showed the higher number of downregulated genes in pathways, such as photosynthesis, antenna protein, carbon fixation in photosynthetic organisms, and porphyrin and chlorophyll metabolism, which ultimately affect the photosynthesis in water dropwort. The genes of hormone signal transduction pathways (ethylene, jasmonic acid, brassinosteroid, and indole-3-acetic acid) showed upregulation in the post-blanched water dropwort plants. Overall, a higher number of genes coding for TFs, such as ERF, BHLH, MYB, zinc-finger, bZIP, and WRKY were overexpressed in blanched samples in comparison with the control. These genes and pathways participate in inducing the length, developmental processes, pale color, and stress tolerance of the blanched stem. Overall, the genes responsive to blanching, which were identified in this study, provide an effective foundation for further studies on the molecular mechanisms of blanching and photosynthesis regulations in water dropwort and other species.

## 1. Introduction

*Oenanthe javanica*, commonly known as water dropwort belongs to the family *Apiaceae*. It is a perennial aquatic vegetable mostly cultivated in several countries, including China, Japan, Korea, and Thailand [1,2,3]. It is a rich source of several vitamins, minerals, dietary fiber, phenolics, and flavonoids. Traditionally, it is also used to treat fever, mumps, jaundice, hypertension, leucorrhea, haematuria, and abdominal pain [1,2,4]. Phytochemicals present in water dropwort, such as persicarin, apigenin, isorhamnetin, quercetin, and hyperoside have different pharmacological activities, such as anticancer, anti-hepatitis B virus (HBV), anti-inflammatory, neuroprotective, hepatoprotective, and antioxidant activities. These nutritional and medicinal properties made water dropwort popular in various countries [1,2,4,5]. It is commonly used in different dishes in East Asian countries because of its distinctive aroma and flavor. In China, people use the steamed stem of water dropwort with boiled rice, and the fried stem of blanched water dropwort is also very popular in China [4,6,7,8].

Blanching is a method used in water dropwort to obtain pale, tender, juicy, and sweet water dropwort stems. This method is used to block sunlight, which ultimately inhibits the chlorophyll production. Consequently, the stem of the water dropwort becomes pale, tender, sweet, and delicious [7,9,10]. The entire water dropwort or its parts are covered in the soil. Different blanching techniques, such as deep planting method, deep water softening method, and soil molding method, are used in processing water dropwort [6,8,10]. The deep planting technique is the most suitable method for water dropwort blanching [11]. Kumar et al. (2021) reported that blanching has a positive effect on the nutritional value and antioxidant capability of the stems of different water dropwort cultivars [7]. Furthermore, water dropwort variety V11E0012 (Jianglingye shuiqin) is best for blanching [7]. However, the basic molecular mechanisms involved in blanching remain unclear. Therefore, the current study’s objective was to reveal the theoretical basis for the molecular mechanisms underlying the photosynthesis related pathways, signal transduction pathway, and transcription factors (TFs) in blanching. For this purpose, we compared the transcriptomes of stem samples of water dropwort at different periods (pre-blanching, mid-blanching, and post-blanching) and control (grown under normal condition in the field) using RNA-sequencing technology. We screened out the pathways and genes that might participate in blanching, tenderness, and photosynthesis regulation. The differential expression of the genes in response to the blanching of water dropwort stem was analyzed, and the potential roles of these transcripts are discussed. This study provides reference data for future research work on water dropwort.

## 2. Results

### 2.1. Transcriptome Sequencing and Assembly

A comparative analysis of water dropwort cultivar V11E0012 (Jianglingye shuiqin) under pre, mid, and post-blanching, as well as control samples was performed. High-quality paired end reads were obtained from the pre-blanching, mid-blanching, post-blanching, and control samples. The percentage of high-quality score (Q30) was >93.50%, GC content was >44.2%, and the mapped ratio was >78.6%. A total of 62,336 unigenes and 224,926 transcripts were generated from de novo transcriptome assembly (Table S2).

### 2.2. Unigene Annotation and Classification

Sequences were subject to the BLAST search against available public databases. The total number of annotated unigenes was 30,666 (Table S2). Nr homologous organism distribution showed that the annotated unigenes hit a range of plant species with sequence identity. *Daucus carota* (82.47%) had the highest identity, and the reason for this high identity was that both plants belonged to the family *Apiaceae*.

### 2.3. Differentially Expressed Genes (DEG) in Water Dropwort Plant in Blanching Conditions

**Figure 1:**
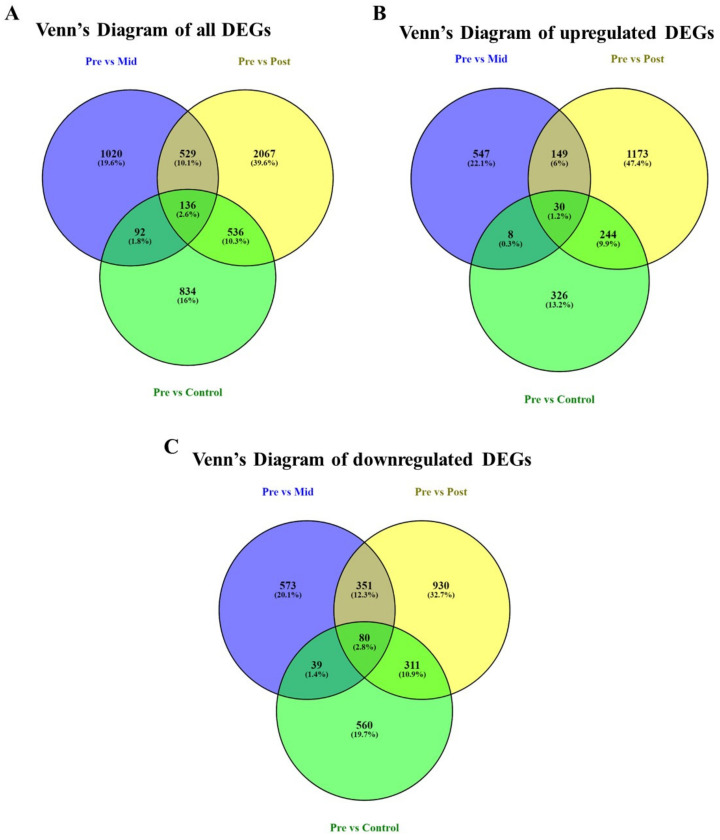
Distribution of differentially expressed genes (DEGs) in the stem of *O. javanica* under blanching treatment. Pre-blanched plants are the reference point for all analyzed samples: mid-blanched, post-blanched plants, and control samples. Blue color represents ‘pre vs. mid-blanching’, yellow represents ‘pre vs. post-blanching’, and green represents ‘pre vs. control’ samples. (**A**) All DEGs, (**B**) upregulated DEGs, and (**C**) downregulated DEGs.

DEGs in the V11E0012 (cultivar of water dropwort) stem samples under pre-, mid-, and post-blanching, as well as those in the control samples, were evaluated. Pre-blanched plants are the reference point for all analyzed samples; mid-blanched, post-blanched, and control. Data revealed that mid-blanched samples have 1777 DEGs compared with pre-blanching samples, in which 734 were upregulated, and 1043 were downregulated. Similarly, post-blanched samples showed 3268 DEGs, including 1596 upregulated and 1672 downregulated DEGs in comparison with pre-blanching samples. Whereas, the control samples have 1598 DEGs, in which 608 were upregulated and 990 were downregulated (Figure S1; Table S3). In the transcriptome analysis, 136 identical DEGs were found in mid-blanching, post-blanching, and control samples. The distribution of upregulated DEGs showed that 30 DEGs were the same in all conditions. A total of 80 downregulated genes were the same in all conditions (Figure 1).

### 2.4. DEGs Functional Annotation

The DEGs were subjected to BLAST search against public databases for functional interpretation. A total of 1662, 2985, and 1493 DEGs were annotated in mid-blanching, post-blanching, and control samples, respectively (Figure S2).

### 2.5. Gene Ontology (GO) Enrichment Analysis

The GO enrichment analysis of the DEGs revealed that post-blanching samples have highest enriched biological process terms. Pre-blanched plants are the reference point to analyzed mid-blanched, post-blanched, and control samples. A higher number of downregulated biological processes terms were found in post-blanched samples, followed by mid-blanched samples. Metabolic process, cellular process, oxidation-reduction process, and transport were the most significantly downregulated terms in post-blanched plants compared with the mid-blanched plants and control samples. Similarly, several other biological processes, such as response to stress, photosynthesis, light stimulus, and chlorophyll biosynthetic process, also showed a higher number of downregulated DEGs in post-blanched samples than the other samples (Table S4).

### 2.6. Kyoto Encyclopedia of Genes and Genomes (KEGG) Pathway Enrichment Analysis

This analysis revealed that 326 DEGs in mid-blanching samples were allotted KEGG IDs and further classified into 96 pathways. Similarly, 614 DEGs in post-blanched samples were allocated KEGG IDs and further classified into 113 pathways. Lastly, 241 DEGs in control conditions were assigned with IDs and further classified into 91 pathways. The most enriched pathways in post-blanched plants in comparison with the mid and control conditions were signal transduction, ribosome, protein processing in endoplasmic reticulum, and starch and sucrose metabolism. Moreover, a higher number of downregulated DEGs post-blanched plants were in ribosome, photosynthesis, carbon metabolism, starch and sucrose metabolism, photosynthesis-antenna proteins, porphyrin and chlorophyll metabolism, and carbon fixation in photosynthetic organisms compared with the mid-blanching and control conditions (Table S5).

#### 2.6.1. Photosynthesis Related Pathways

**Figure 2:**
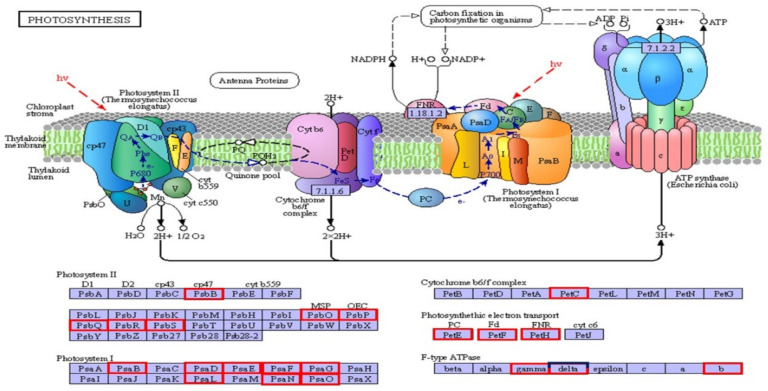
DEGs involved in photosynthesis pathway of blanched (post-blanching) stem of water dropwort. Red color boxes indicate the downregulated genes, and the blue color represents the upregulated gene [12,13].

Photosynthesis related genes in the pathways of photosynthesis, antenna proteins, carbon fixation, and porphyrin and chlorophyll metabolism were suppressed in post-blanched plants in companion with pre-blanched plants. In the photosynthesis pathway, we found 36 DEGs in post-blanching samples, among which 35 were suppressed. The only upregulated gene was *c47691.graph_c1* which codes for the *delta* subunit of F-type ATPase complex (Figure 2). Similarly, 32 DEGs were found in mid-blanching samples, and all were downregulated. Furthermore, control samples showed six DEGs, in which two were upregulated.

#### 2.6.2. Photosynthesis-Antenna Proteins Pathway

**Figure 3:**
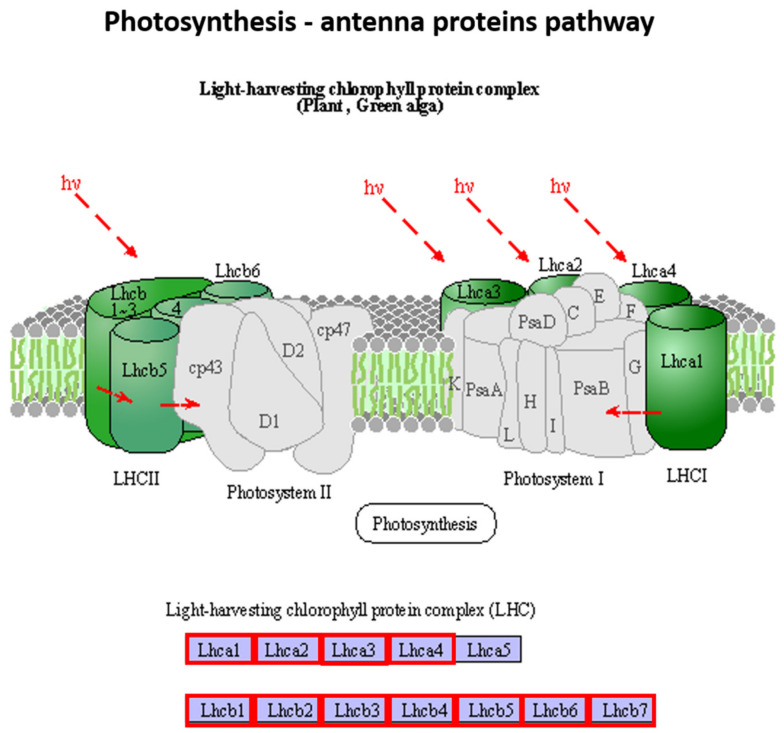
DEGs involved in photosynthesis-antenna proteins pathway of the blanched stem (pre vs. post-blanching) of water dropwort. Red color boxes indicate the downregulated genes [12,13].

In this pathway, 19 DEGs were identified in post-blanching samples, and all were downregulated (Figure 3). Similarly, 18 DEGs were identified in mid-blanching samples, and no gene was upregulated among them. There is only one gene of decreased expression in the control plant.

#### 2.6.3. Carbon Fixation in Photosynthetic Organisms

**Figure 4:**
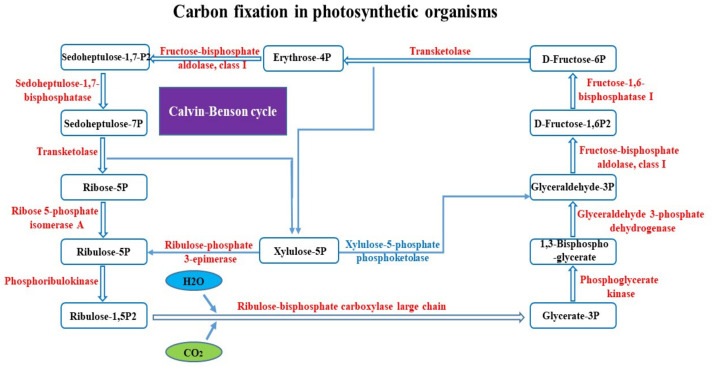
DEGs involved in “carbon fixation pathway in photosynthetic organisms” from blanched stem of water dropwort (post-blanching sample).

We found 18 DEGs in post-blanching samples, among which 16 were suppressed (Figure 4). The two upregulated DEGs were *c17894.graph_*c0 (*Ribulose bisphosphate carboxylase small chain 1*) and *c44566.graph_c2* (*Chloroplastic glutamate–glyoxylate aminotransferase 2*). On the other hand, 13 DEGs in mid-blanched samples and 8 DEGs in control samples were found suppressed.

#### 2.6.4. Porphyrin and Chlorophyll Metabolism Pathway

**Figure 5:**
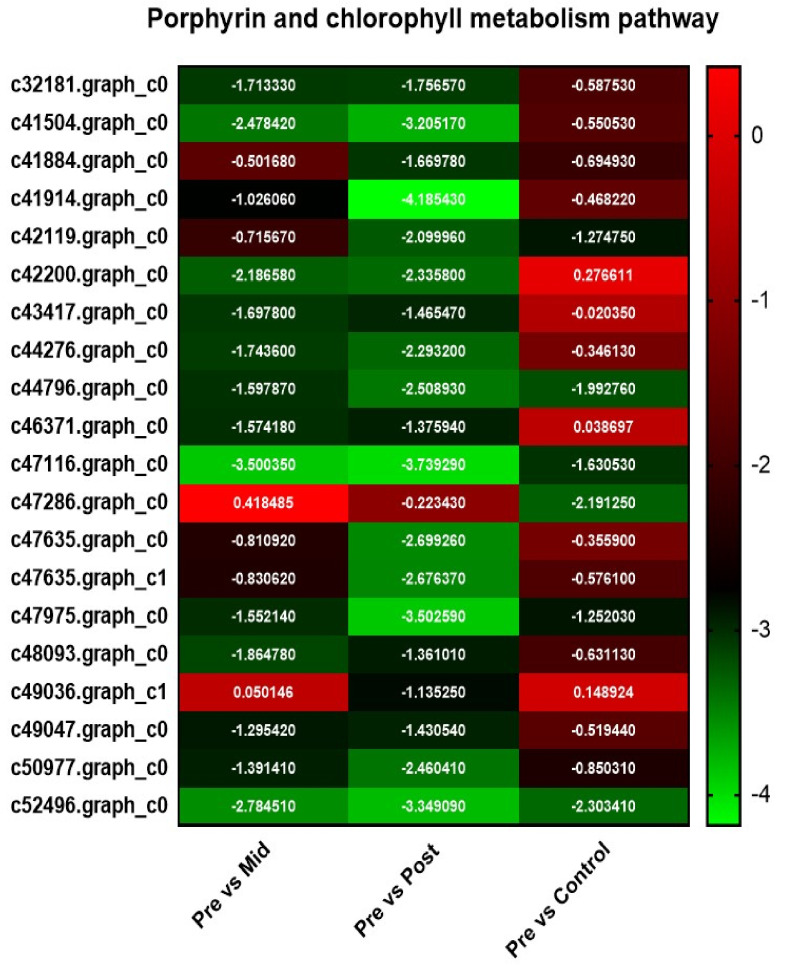
Heat map of DEGs of porphyrin and chlorophyll metabolism pathway in the water dropwort stem under ‘pre vs. mid–blanching’, ‘pre vs. post–blanching’, and ‘pre vs. control’ conditions. Scale represents the expression level of genes ranging from green (downregulation) to red (upregulation).

The current study showed that 17 genes in the porphyrin and chlorophyll metabolism pathway were downregulated in post-blanched samples in comparison with pre-blanched samples. Similarly, 12 DEGs in mid-blanched and 4 genes in control samples were downregulated in this pathway. No gene was found upregulated in any case. The relative expression of the DEGs is presented in Figure 5.

#### 2.6.5. Plant Hormone Signal Transduction Pathway

In this pathway, we found 48 DEGs in post-blanched samples, among which 32 were upregulated and 16 were downregulated. 20 genes were dysregulated in mid-blanched samples, 13 of which were upregulated, and 7 were downregulated. The control sample showed the presence of 13 upregulated and 5 downregulated genes.

**Figure 6:**
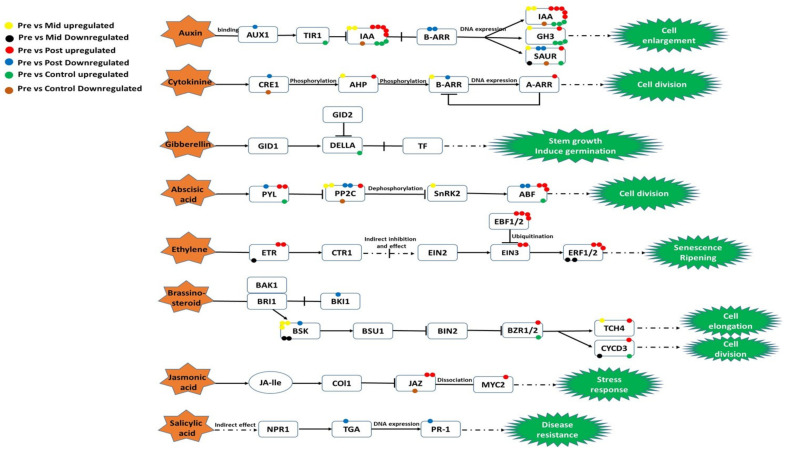
DEGs involved in plant hormone signal transduction pathways in the stem of water dropwort. Yellow color balls represent upregulated DEGs of ‘pre vs. mid-blanching’ and black color represents downregulated DEGs of ‘pre vs. mid-blanching’. Red color balls represent upregulated DEGs of ‘pre vs. post-blanching’, the blue color represents downregulated DEGs of ‘pre vs. post-blanching’, green color balls represent upregulated DEGs of ‘pre vs. control’ samples, and the brown color represents downregulated DEGs of ‘pre vs. control’.

We found seven upregulated and five downregulated genes involved in the auxin signaling pathway of auxin synthesis in the post-blanched samples. Similarly, four genes were upregulated in mid-blanched samples, and the control samples possessed eight upregulated and three downregulated genes related to auxins. The two upregulated and two downregulated genes of cytokinine (CTK) were detected in post-blanched samples; two genes were upregulated in mid-blanched samples; and only one gene was downregulated in the control samples (Figure 6). Gibberellin (GA) showed only one dysregulated gene, and this gene was upregulated in the control samples. The number of upregulated genes related to abscisic acid (ABA) were six, three, and two for post-blanched, mid-blanched, and control samples, respectively. Moreover, five DEGs were downregulated in the post-blanched samples, and one gene was downregulated in the control samples (Figure 6).

Post-blanched samples showed seven upregulated genes related to ethylene. However, three downregulated genes related to ethylene were found in mid-blanched samples. The number of upregulated genes related to brassinosteroid (BR) in post-blanched, mid-blanched, and control samples were three, four, and two, respectively. By contrast, two downregulated genes related to BR in post-blanched and three in mid-blanched were detected. We found three upregulated genes related to jasmonic acid (JA) in post-blanched plants, and only one downregulated gene was found in the control samples. Furthermore, only two downregulated genes related to salicylic acid were observed in the post-blanched samples (Figure 6).

### 2.7. Transcription Factors

**Figure 7:**
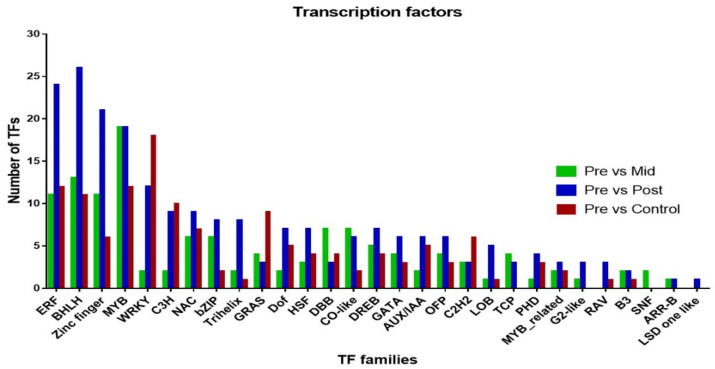
Distribution of transcription factors DEG from three analyses: plants before blanching versus mid–blanching; plants before blanching versus post–blanching; and plants before blanching versus control conditions.

This study depicted that 215 TFs were differentially expressed in post-blanched samples (150 were upregulated and 65 were downregulated) and were categorized into 28 families. The most enriched families were bHLH that have 17 upregulated and 9 downregulated DEGs, followed by ERF which have 22 upregulated and 2 downregulated DEGs, zinc finger has 14/7, MYB has 17/2, and WRKY has 6/6 DEGs (Figure 7; Table S5). In comparison, 127 TFs were differentially expressed in mid-blanched samples (47 upregulated and 80 downregulated). These TFs were categorized into 27 different families, and the most enriched families were MYB that has 8 upregulated and 11 downregulated DEGs, followed by bHLH that has 2 upregulated and 11 downregulated DEGs, ERF has 5/6, zinc finger has 5/6, and DBB has 0/7 DEGs. Furthermore, 132 TFs were differentially expressed in control samples (44 genes upregulated and 88 downregulated), and these TFs were further categorized into 24 families. The most enriched family was WRKY, which has 18 downregulated DEGs, followed by ERF that has 2 upregulated and 10 downregulated DEGs; MYB has 7/5, BHLH has 2/9, and C3H has 6/4 DEGs (Figure 7; Table S6).

### 2.8. Validation of DEGs

**Figure 8:**
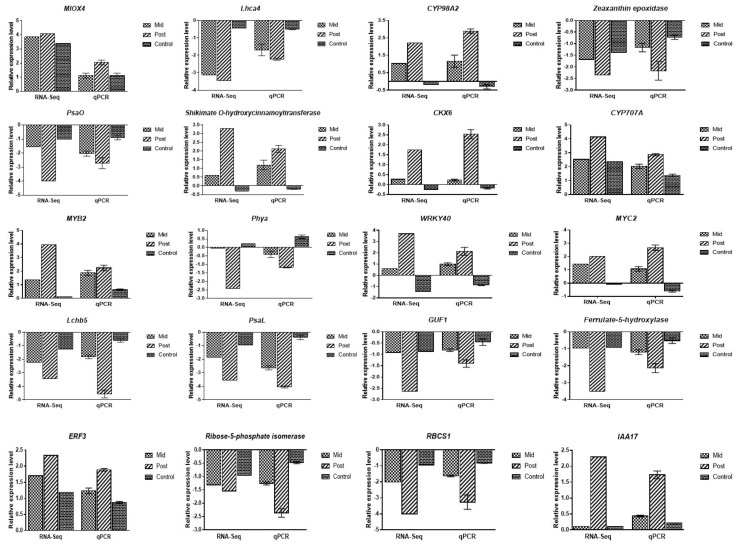
qRT–PCR of selected genes for the validation of RNA-seq data in mid-blanching, post-blanching, and control conditions. The black bars on the qPCR data represent the means ± SD.

qRT-PCR analysis was conducted on 20 selected genes from different pathways to validate the RNA-seq data; control, pre-, mid-, and post-blanching were used in the qRT-PCR analysis. The results showed that the expression profiles of selected genes under qRT-PCR were in agreement with the results obtained from the RNA-seq, which indicates the accuracy and reproducibility of the RNA-seq data (Figure 8).

## 3. Discussion

### 3.1. Blanching Affects the Light-Dependent and Light-Independent Reactions of Photosynthesis in Water Dropwort

Light is the driving force of photosynthesis and provides a signal for plant morphological and physiological adaptions under various environmental conditions [14]. Different studies showed that in etiolation conditions, photosynthesis rates are significantly reduced compared with the control samples [15,16,17]. Moreover, photosynthesis regulation is controlled by the activities of photosynthesis-related enzymes [14,16,17]. Previously, Kumar et al. (2021) found that chlorophyll content is remarkably decreased under blanching conditions, particularly in post-blanching samples [7]. The main reason behind this occurrence is the absence of light due to deep cultivation. Plants have higher chlorophyll content and possesses more light-harvesting antennae under natural conditions than in blanching conditions. The genes expression involved in photosynthetic pathways influences chlorophyll content [15,16,17].

The photosystem II (PSII) is surrounded by light-harvesting complex proteins (LHCPs), including major and minor LHCP II, and form complexes with chlorophyll and xanthophylls [18]. After blanching, the photosynthetic systems of water dropwort showed altered expression profiles. The present study revealed the downregulation of the “light-harvesting complex (LHC) components” used in trapping and transporting light energy to PSI and PSII, such as *RPS4* (*c39632*.graph_c0), *Lhca4* (*c40818.graph_c0*), and *Lhca1* (*c42181.graph_c0*). Similarly, other genes, such as*Lcha2-3* and *Lhcb1-7*, were also downregulated in the mid- and post-blanching samples. The only gene downregulated in the control samples was *Lhcb4*. The results of previous studies also reported that chlorophyll levels decreased in the etiolated shoots of *Olea europaea* and *Juglans regia* [17,19]. *The Lhc* genes were also reported to be downregulated under etiolated conditions. Etiolated *Brassica rapa* leaves also showed a repression in *Lhc* genes [15]. Plants with repressed *Lhcb1* and *Lhcb2* genes are pale green and have low chlorophyll content [18,20]. The low chlorophyll content and pale color of water dropwort could be due to the downregulation of these *Lhc genes* [21,22].

In the current study, PSII components, such as *psbB* (*c51941.graph_c0*), *psbO* (*c47984.graph_c0*), *psbR*, *psbP*, *psbQ*, and *psbS* were found to be downregulated. Similarly, *psaB* (*c53042.graph_c0*), *psaN* (*c43347.graph_c0*), *psaO* (*c41978.graph_c0*), *psaD-G*, and *psaL* in PSI were also downregulated. Wang et al. (2020) also reported the downregulation of PSI genes (*psbB*, *psbO*, *psbR*, *psbP*, *psbQ*, and *psbS*) and PSII genes (*psaB*, *psaD-G*, *psaL*, *psaN*, and *psaO*) in the etiolated cotyledons of *Cunninghamia lanceolate* [23]. Furthermore, the genes for components of cytochrome b6f complex, such as *petC* (*c37658.graph_c0* and *c38988.graph_c0*); photosynthetic electron transport, such as *petE* (*c30570.graph_c0*), *petF* (4 genes), and *petH* (*c48071.graph_c0*); and F-type H^+^-transporting ATPase subunits *gamma* (*c39370.graph_c0*), *delta* (*c43471.graph_c0*), and *b* (*c37746.graph_c0*) were also found to be downregulated in mid and post-blanching samples. They are involved in electron transport between PSII and PSI, and used for ATP formation [24]. The earliest result is also in agreement with the current study, which showed *petE*, *petF*, *petH,* and F-type ATPase subunit *gamma*, *delta*, and *b* were downregulated under dark conditions [23]. Therefore, we speculate that alteration in the expression of genes involved in PSI and PSII may be responsible for the etiolated stem.

Apart from these findings, blanching also inhibits carbon assimilation by impeding the Calvin–Benson cycle. When the water dropwort was subjected to blanching, reduction in gene expression encoding ribulose-1, 5-bisphosphate carboxylase/oxygenase (Rubisco) was detected. Gene encoding the Rubisco subunit (*c44466.graph_c0*) was repressed in both mid- (logFC −2.01695) and post-blanching (logFC −4.00885) conditions. These results agree with previous studies where a lower level of Rubisco protein was found in the etiolated leaves of *Brassica rapa* [15]. The expression of the other 13 genes in post-blanched samples and 10 genes mid-blanched samples were found to be downregulated, such as *GAPDH*, *fructose-bisphosphate aldolase*, *FBPase*, *transketolase*, and *SBPase*, *ribose-5-phosphate isomerase*, *ribulose-phosphate 3-epimerase*, and *phosphoribulosekinase* (*PRK*). By contrast, the control samples have only five downregulated genes that encode the enzymes of the Calvin–Benson cycle. According to Michelet et al. (2013), four enzymes of the Calvin–Benson cycle *PRK*, *GAPDH*, *FBPase*, and *SBPase*, were downregulated under dark conditions [25], which is in agreement with the current study. The suppression of these genes indicated that blanching shut down this energy generating process. The result indicates that the pathways associated with processes related to photosynthesis were largely suppressed, which could be a retrograde process regulated by organelle-to-nucleus signaling [15].

Yang et al. (2016) showed that the porphyrin and chlorophyll metabolism, as well as carotenoid biosynthesis play an important role in etiolation phenotype [21]. Similarly, Lyu et al. (2017) showed that the downregulated genes encoding *chlorophyllide-a oxygenase* involved in porphyrin and chlorophyll metabolism and *abscisic-aldehyde oxidase* might cause the difference in leaf color [26]. In this study, we detected the downregulation of 17 genes in post-blanched samples and 12 genes in mid-blanched samples involved in the porphyrin and chlorophyll metabolic pathway, such as *chlorophyllide-a oxygenase*, *magnesium chelatase*, *protochlorophyllide reductase,* and *uroporphyrinogen decarboxylase*. A study on *Brassica rapa* showed that *protochlorophyllide reductase* has an important role in Pchlide homeostasis and the greening of etiolated plants. They also found the downregulation of *protochlorophyllide reductase* in etiolated conditions [15]. Furthermore, we also found genes encoding important enzymes, including *abscisic-aldehyde oxidase*, *zeaxanthin epoxidase*, and *(+)-abscisic acid 8′-hydroxylase,* which were downregulated and participated in the carotenoid biosynthesis pathway. These genes of porphyrin and chlorophyll metabolism and carotenoid biosynthesis pathways change the pale color of water dropwort and might have an important role in the blanching of water dropwort stem. However, functional genomics is needed for confirmation.

### 3.2. Plant Hormone Signal Transduction Pathways

In this transcriptomic analysis, several genes in the plant hormone signal transduction pathways in the stem of water dropwort were examined in response to blanching. Different hormones, including ethylene, ABA, auxin, BR, CTK, JA, GA, and salicylic acid, showed distinct regulation patterns.

Auxin is used to stimulate organogenesis and patterning in plants [22]. *PIN* and *PILS* show a significant role in auxin accumulation in developing organs, and the upregulation of these genes are positively associated with IAA accumulation [27,28]. In the current study, *PIN*, *SAUR*, *GH*, and *IAA* were upregulated in the blanched stem, which agrees with the previous finding that the level of *PIN* increased under low light [29]. We also found several downregulated genes in the post-blanched samples that encode AUX1, ARR-B, and SAUR. *IAA1* is induced by auxin under light stress and helps in the elongation of coleoptile in rice [22]. We also found *IAA1* upregulation in blanched samples, and *IAA1* downregulated in the control samples. The concentration of IAA can induce the etiolated stem length [17]. These findings indicate that the upregulation of *IAA* and *PIN* helps plants during the blanching process to tolerate stress and induce the length of the blanched stem.

GA is another hormone that has a role in plant growth [30]. In this study, the control samples revealed the upregulation of *DELLA* and *GA20ox* genes in comparison with pre-blanched samples. *GA20ox* helps promote GA accumulation [31,32]. *GA20ox* was downregulated under mid- and post-blanching conditions. Weller et al. (2009) also reported that GA level is affected under dark conditions [33]. These results indicate that GA improves vegetative growth under normal conditions but not in the blanched stem of water dropwort. CTK is a hormone that assists in regulating cell proliferation and tissue development in plants [34]. In the current study, we found that *CKX* was upregulated under blanching conditions and downregulated in the control samples. Carabelli et al. (2007) mentioned that *CKX*6 expression is increased under the partial absence of light [35], and a higher CTK level was observed in the young tissues of *Elaeocarpus hookerianus* and *Prunus persica* [36,37]. Furthermore, this result also showed the role of CTK in the plant response to blanching which results in stem rejuvenation. ABA is used as a negative controller of plant growth which contributes to the transition of *Pinus pinea* and *Pinus radiate* from the vegetative stage to mature stages [38,39]. *CYP707A* and *PYL* regulate ABA signal transduction in ABA biosynthesis. In the current study, *CYP707A* and *PYL* were upregulated under blanching conditions, and the difference between the blanched plant and the control one increased with the increasing duration of blanching. This result agrees with the published results that the expression of *CYP707A* and *PYL* genes in the etiolated shoots of walnut is elevated [17]. Our finding implied that the upregulation of the genes involved in ABA signal transduction participates in regulating the blanching of water dropwort. Ethylene is an important phytohormone that plays a positive role in various plant developmental processes and can assist plants under different abiotic stress conditions [40]. The genes of the EIN3/EIL family are involved in regulation and activation of other transcription factors, such as *ERF1* regulating the genes expression in response to ethylene [41,42,43]. In this study, DEGs encoding ETR, EBF1-2, and EIN3 were found upregulated, and *CTR1* was downregulated in blanched samples. *ERF* are important genes that participate in signal transduction. A total of 22 *ERF* genes were upregulated in the post-blanching samples, whereas only two *ERF* genes were upregulated in the control samples. Previous studies have shown that the level of ethylene was also increased in the stems under dark conditions [17]. Previous studies have reported that *LeERF1* positively modulated the ethylene response on etiolated seedling, plant development, and softening in tomato [44]. Lu et al. (2017) reported that ethylene causes the chlorophyll degradation in the peels of two citrus species [45], indicating that etiolation may increase ethylene in tissues. In the current study, we found that upregulation of many ethylene related genes in the blanched stems of water dropwort, and we speculate that it has an important role in response to blanching conditions, plant developmental processes, and pale coloration of stem. Another hormone is BR, which participates in plants growth and development, and contributes to the regulation of photomorphogenesis [46]. In the present study, the *CYP90* gene involved in BR biosynthesis was upregulated in the post-blanching and control samples, but the level of its expression was higher in the blanched sample. A previous study also showed the same results and mentioned that *CYP90* expression increases in low light and in the partial absence of light [47]. Therefore, we speculate that BR could play a role under blanching condition on photomorphogenesis and stress tolerance. Jasmonic acid (JA) is a growth regulating substance also involved in the response to abiotic and biotic stresses [48]. In the present study, JA biosynthesis-related genes (*JAZ* and *MYC2*) were upregulated in the blanched stem of water dropwort. According to Zhang et al. (2015), *JAZ* and *MYC2* are upregulated in response to environmental stress [49]. Another study also showed that JAZ–MYC has a noteworthy part in the JA signaling pathway through the integration of TFs, phytohormones (ABA, JA, SA, GA, IAA, and ET), and related genes [48]. Concurrently, JA has synergistic and antagonistic effects with other plant hormones, such as ABA, ethylene, and salicylic acid, in response to different environmental stress [48]. Therefore, JA has a significant role in the response of water dropwort to blanching.

Overall, a high number of DEGs promoting the biosynthesis of hormones, such as IAA, BR, JA, and genes of the pathways related to ethylene synthesis showed an increased expression in blanched plants. The results suggest that blanching could induce hormone signal transduction pathways in the stem of water dropwort. The pale color and increased stem length are speculated to be due to the positive regulation of these hormones to related genes [25,32,50].

### 3.3. Transcription Factors

TFs are used to regulate the expression of different genes. Several TFs, such as ERF, BHLH, zinc finger, MYB, WRKY, bZIP, and NAC, have been identified in response to biotic and abiotic stresses [50,51,52,53,54,55,56]. ERFs are involved in response to environmental stress [57]. *ERF1* could be induced by different abiotic and biotic stresses. *LeERF1* is found to be involved in induction of etiolated seedling, plant development, and softening in tomato [44]. Similarly, *ERF4* could also be stimulated through ethylene and JA [58,59] and play an important role in blanching as mentioned earlier. We found 22 upregulated ERF genes, including *ERF1* and *ERF4* in the blanched stem of water dropwort, whereas the control showed only two upregulated genes. The bHLH and bZIP TF families play a role in photomorphogenesis and act as regulators in anthocyanin biosynthesis [60,61]. *HY5* is a bZIP-TF is used to enhances the expression level of light-inducible genes, which can lead to *HY5* photomorphogenesis [62]. Previous reports showed the higher expression of *HY5* under the dark condition in comparison with light exposure. The interaction of *COP1*-*SPA1* with *PHYTOCHROME A* (*PHYA*) signaling plays a vital part in *HY5* degradation, which ultimately causes the repression of photomorphogenesis in dark conditions [16]. We found *HY5* upregulation and *PHYA* downregulation in the blanched stem of water dropwort. Numerous bHLHs, including R2R3-MYBs, showed a higher expression level in etiolated leaves [15]. In this study, we also found 17 upregulated bHLH genes in the blanched stem. However, only two upregulated bHLH genes were observed in the control samples. Thus, these genes might have a vital role in the blanching of water dropwort. Similarly, the expression of *MYB12* and *MYB111* was induced under UV-B light [63]. Furthermore, *MYB15* upregulation was observed under dynamic light conditions compared with the control conditions [14]. In the present study, we found 17 upregulated MYB genes in the blanched samples, including *MYB2*, *MYB4*, *MYB102*, and *LIMYB*, which might play a role in response to blanching conditions in water dropwort. WRKY TFs, such as *WRKY76*, act as a positive regulator for submergence and drought tolerance [64]. The expression level of *WRKY76* in sunflower leaves was induced under drought and reaeration following submergence [65]. Our study found six upregulated WRKY genes in blanched water dropwort, whereas the control samples showed 18 downregulated genes of WRKY. There is probability that the control plants are 40 days older than the pre-blanched plants, resulting in more WRKY genes with decreased expression. Similarly, we also found a higher number of upregulated genes related to zinc-finger, bZIP, C3H, NAC, Trihelix, dof, HSF, LBD, and AUX/IAA in the blanched stems of water dropwort in comparison with the control samples. The upregulation of most TFs suggests that these TFS have an important role in blanching and promotes phytohormone synthesis and signal transduction, as well as polyphenols and flavonoid biosynthesis pathways.

## 4. Material and Methods

### 4.1. Experimental Conditions

For this study, water dropwort cultivar V11E0012 (Jianglingye Shuiqin) was grown in fertile soil with an appropriate irrigation level. The mature stems of water dropwort were sliced into 3.3–3.5 cm segments, and every segment contained a stem node. The cut stems were placed in a ventilated and low-temperature area, and sunshade nets were installed for moisture retention. Water was sprinkled each day, and new shoots developed after 78 days. These new shoots were transferred to seedbeds and covered with a thin layer of soil; then a sunshade net was used to cover the seedbeds. After 1 month, the plants with approximately 10 cm height were planted with the hill planting method, that is, the space between hills was 10 cm × 10 cm, and each hill had three or four plants. Finally, blanching was performed when the plants’ height was approximately 30 cm [7,9,66].

### 4.2. Deep Planting Technique Used for Blanch Cultivation

Water dropwort with a height of approximately 30 cm were bound in bunches and placed for 40 days in 2022 cm deep soil [9,66]. Stem samples were collected at four time points: pre-blanching (before blanching), mid-blanching (blanched for 20 days), post-blanching (blanched for 40 days), and control (grown under normal conditions in the field for 40 days). All experiments were performed in triplicate.

### 4.3. RNA Extraction, Library Construction, and Illumina Sequencing

RNA from the stems of the control, pre-blanched, mid-blanched, and post-blanched samples were extracted with Trizol (Invitrogen, Santa Clara, CA, USA). The quality and quantity of RNA for transcriptome sequencing were measured with BioDrop uLite (80-3006-51).

A total amount of 1 µg RNA per sample was used as the input material for the RNA sample preparations. Sequencing libraries were generated using NEBNext^®^Ultra™ RNA Library Prep Kit for Illumina^®^ (NEB, Ipswich, MA, USA) following the manufacturer’s recommendations and index codes were added to attribute sequences to each sample. Briefly, mRNA was purified from total RNA using poly-T oligo-attached magnetic beads. Fragmentation was carried out using divalent cations under an elevated temperature in NEBNext first-strand synthesis reaction buffer (5×). First-strand cDNA was synthesized using random hexamer primer and M-MuLV Reverse transcriptase. Second strand cDNA synthesis was subsequently performed using DNA Polymerase I and RNase H. The remaining overhangs were converted into blunt ends via exonuclease/polymerase activities. After adenylation of 3′ ends of DNA fragments, NEBNext adaptor with hairpin loop structure were ligated to prepare for hybridization. In order to select cDNA fragments of preferentially 240 bp in length, the library fragments were purified with AMPure XP system (Beckman Coulter, Beverly, CA, USA). Then, 3 µL USER Enzyme (NEB, Ipswich, MA, USA) was used with size-selected, adaptor-ligated cDNA at 37 °C for 15 min followed by 5 min at 95 °C before PCR. After this, PCR was performed with Phusion High-Fidelity DNA polymerase, Universal PCR primers, and Index (X) Primer. At last, PCR products were purified (AMPure XP system) and library quality was assessed on the Agilent Bioanalyzer 2100 system. The clustering of the index-coded samples was performed on a cBot Cluster Generation System using TruSeq PE Cluster Kit v3-cBot-HS (Illumia) according to the manufacturer’s instructions. After cluster generation, the library preparations were sequenced on an Illumina Hiseq 2000 platform and paired-end reads were generated [3,67].

### 4.4. Quality Control and Transcriptome Assembling

Raw data (raw reads) in fastq format were first processed through in-house perl scripts. In this step, clean data (clean reads) were obtained by removing reads containing adapter, reads containing poly-N, and low-quality reads from raw data. The Q20, Q30, GC-content, and sequence duplication level of the clean data were calculated. All the downstream analyses were based on clean data with high quality. The left files (read1 files) from all libraries/samples were pooled into one large left.fq file, and right files (read2 files) were pooled into one large right.fq file. Transcriptome assembly was accomplished based on the left.fq and right.fq using Trinity with min-kmer-cov set to 2 by default and all other parameters set to default [3,67]. Transcriptomic data was submitted to the Sequence Read Archive (SRA) database of the National Center for Biotechnology Information (SRA accession number: PRJNA722062).

### 4.5. DEG Identification and Functional Annotation

The expression levels of genes were calculated through RNA-seq by expectation maximization for every sample [68]. The gene expression analysis of the four conditions was accomplished with the DESeq R package (1.10.1). Pre-blanched plants are the reference point to analyze mid-blanched, post-blanched, and control samples. For significant differential expression, *p*-value < 0.05 and Log2 (fold change) > 1 were fixed as the threshold [3,67]. GO and pathways enrichment analyses of the DEGs were performed by topGO R packages and KOBAS software, respectively [69,70]. Transcription Factor Database PlnTFDB and PlantTFDB were used in the identification of dysregulated TFs [3].

### 4.6. Quantitative Real-Time PCR Analysis

RNA from the stems of the control, pre-blanching, mid-blanching, and post-blanching samples were extracted with Trizol (Invitrogen, Santa Clara, CA, USA). Reverse transcription reactions were performed using SuperScript III reverse transcriptase (Invitrogen, Grand Island, NY, USA) according to the manufacturer’s instructions. cDNA synthesis was performed using oligo(dT)_20_ primers. CFX96 real-time PCR system and Bio-rad with SYBR Premix Ex Taq™ II (Tli RNaseH Plus) (TaKaRa Biotech. Co.) were used in the RT-qPCR assays. Primer3 software (https://primer3.ut.ee/ accessed on 23 November 2020) was used in primer design. In this study, we used Actin (*c41123.graph_c0*) as the reference gene. The primers used for the 20 selected genes and Actin are highlighted in Table S1. The amplification protocol of Kumar et al. (2020) was used [3]. qRT-PCR detection was performed in three biological replicates. The relative expression levels were estimated with the 2^−ΔΔCt^ method [71].

## 5. Conclusions

In this study, the transcriptomic response to blanching of water dropwort stem are presented based on GO terms, KEGG functional enrichment analyses, and PlantTFDB analyses. The generated data provides a basis for further studies on the transcriptomic response to blanching and photosynthesis in water dropwort and other related species. Several molecular mechanisms, such as photosynthesis, phytohormones and signaling, and TFs were highlighted in response to blanching. The differential expression of highlighted genes and pathways might be important in many aspects of the plant stress response and photosynthesis regulations. However, further research is still needed to confirm the results. This study will provide reference data for future research on water dropwort.

## Data Availability

The datasets generated and analyzed during the current study are available in the National Center for Biotechnology Information (NCBI) repository, PRJNA722062 (https://www.ncbi.nlm.nih.gov/bioproject/PRJNA722062, accessed on 1 November 2021).

## Supplementary Materials

The following are available online at https://www.mdpi.com/article/10.3390/plants10112484/s1, Figure S1: Volcano plot of differentially expressed genes (DEGs) in the stem of water dropwort under blanching treatment, Figure S2: Functional annotations of DEGs against public databases, Table S1: Information of the qRT–PCR primers, Table S2: transcriptomic assembly data, Table S3: DEGs in all treatments, Table S4: Enriched biological processes in GO analysis of DEGs, Table S5: KEGG analysis of DEGs, and Table S6: TFs involved in blanching.
