# Supplementary material for: Comparative Transcriptomic Analysis Provides Novel Insights into the Blanched Stem of Oenanthe javanica
Source: Plants (Basel). 2021 Nov 17;10(11):2484. doi: 10.3390/plants10112484 (PMC8625949; doi:10.3390/plants10112484)
Supplement: Supplementary file 1 [file plants-10-02484-s001.zip › Supplementary Figures.pdf]

Supplementary Figures

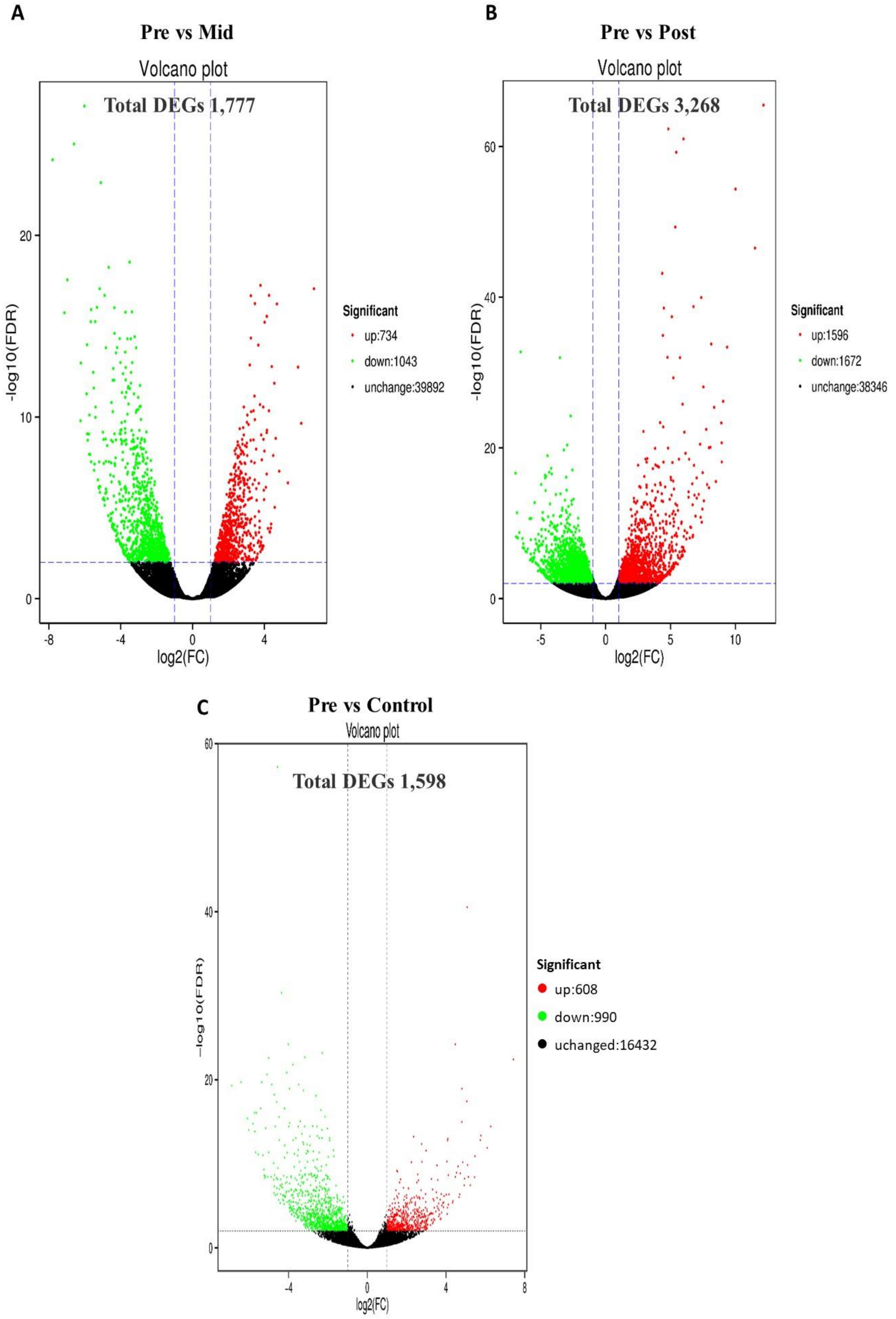

Figure S1. Volcano plot of differentially expressed genes (DEGs) in the stem of water dropwort under blanching treatment. A) Pre vs mid-blanching, B) pre vs post-blanching, and C) pre vs control conditions.

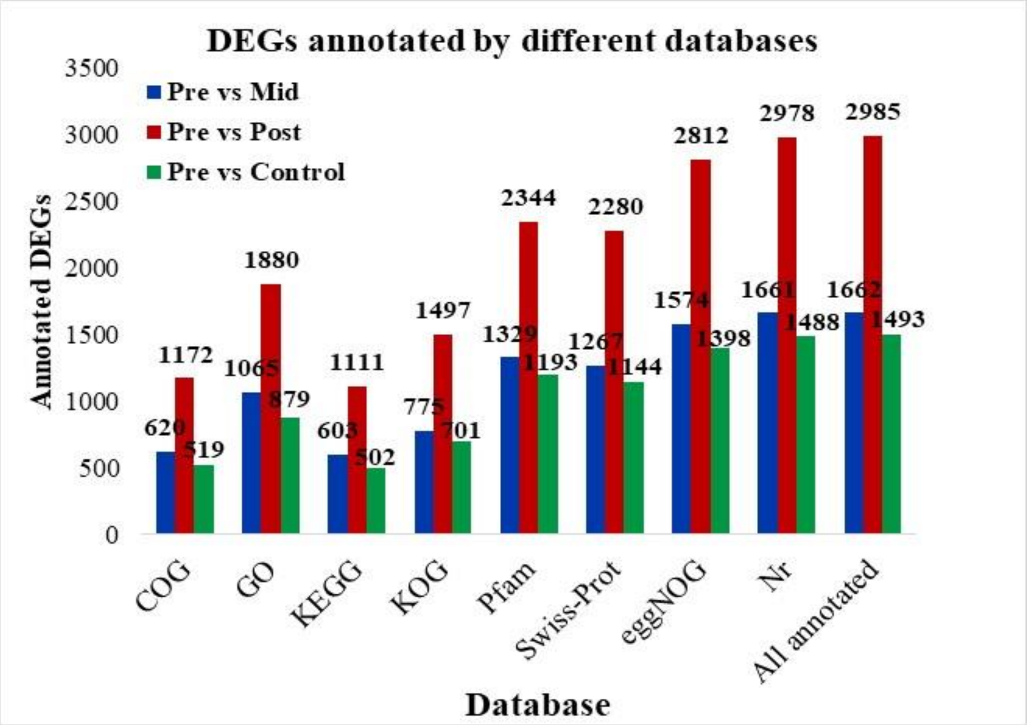

Figure S2. Functional annotations of DEGs against public databases.
